# Approach avoidance training versus Sham in veterans with alcohol use disorder: protocol for a randomized controlled trial

**DOI:** 10.1186/s12888-023-04961-z

**Published:** 2023-07-12

**Authors:** M. M. Caudle, R. Klaming, C. Fong, K. Harlé, C. Taylor, A. Spadoni, J. Bomyea

**Affiliations:** 1 San Diego Joint Doctoral Program in Clinical Psychology, San Diego State University, University of California, 6363 Alvarado Court, Suite 103, San Diego, CA 92120 USA; 2 Department of Veteran Affairs Medical Center, 3350 La Jolla Village Dr, San Diego, CA 92161 USA; 3 Department of Psychiatry, University of California San Diego, 9500 Gilman Dr, La Jolla, San Diego, CA 92093 USA; 4 VA San Diego Center of Excellence for Stress and Mental Health, 3350 La Jolla Village Dr, San Diego, CA 92161 USA

**Keywords:** Alcohol use disorder, Veterans, Approach Avoidance Training, Functional magnetic resonance imaging, Randomized controlled trial, Outpatient, Cognitive bias modification

## Abstract

**Background:**

Alcohol use disorder (AUD) is highly prevalent and commonly co-occurs with other psychiatric disorders among Veterans. Provisional evidence supports the use of Approach Avoidance Training (AAT) - a form of computer-delivered cognitive bias modification designed to target implicit approach bias for alcohol-related cues - as an adjunctive program to treat AUD. However, the extent to which AAT is effective for improving AUD recovery outcomes in outpatient Veteran samples and those with psychiatric comorbidities has been understudied to date. Here we describe a double-blind randomized controlled trial of AAT versus a comparison condition (Sham) being conducted in Veterans with comorbid psychiatric conditions completing outpatient standard care.

**Methods:**

One hundred thirty-six Veterans currently receiving outpatient treatment for AUD will be recruited for this randomized controlled trial with parallel group assignment. Participants will be randomized to either 6 weeks of AAT (n = 68) or Sham (n = 68) training in conjunction with usual care. Assessments will occur at baseline and 6 weeks, 3 months, and 6 months post-baseline. Primary outcome variables will include functional consequences of drinking. Secondary outcome variables will include alcohol consumption, and behavioral indicators of alcohol approach bias. A subset of participants (n = 51) will also complete functional magnetic resonance imaging (fMRI) to assess neural response during an alcohol approach bias assessment.

**Discussion:**

This study is the first randomized controlled trial of AAT administered as an adjunctive treatment to standard care in Veterans with AUD and comorbid psychiatric disorders. Additionally, behavioral and neuroimaging data will be used to determine the extent to which AAT targets approach bias for alcohol cues. If effective, AAT may be a promising low-cost adjunctive treatment option for individuals with AUD.

**Registry name:**

AAT for Alcohol Use Disorder in Veterans.

**Trial registration:**

ClinicalTrials.gov: NCT05372029; Date of Registration: 5/9/2022.

**Supplementary Information:**

The online version contains supplementary material available at 10.1186/s12888-023-04961-z.

## Background

Alcohol use disorder (AUD) is the most common substance use disorder among Veterans [1, 2] and results in substantial functional impairment and adverse outcomes spanning poor health, under or unemployment, legal consequences, financial stressors, and social disruption [3–6]. Psychiatric comorbidity is frequent among Veterans with AUD, with estimates of co-occurring psychiatric disorders exceeding 30% [7, 8], which often further exacerbates functional problems and is associated with increased relapse rate and earlier relapse [9]. Psychosocial (e.g., Cognitive Behavioral Therapy [CBT]) and pharmacotherapies are considered frontline treatments for both AUD and associated psychiatric disorders, yet relatively high relapse rates point to a continued need to identify novel clinical targets to improve outcomes [10–16].

Dual process models of AUD propose that problematic use arises from an imbalance of two conflicting, partially independent sets of processes that could be targeted clinically [17–20]. The first is relatively diminished functioning of top-down, reflexive control systems [19], associated with self-regulation and goal-directed behavior, supported by frontoparietal neural circuits (e.g., dorsolateral and ventrolateral prefrontal cortex (dLPFC; vPFC) and anterior cingulate (ACC)). The second is an over activation of bottom-up, impulse-based responding rooted in mesocorticolimbic circuitry (e.g., medial prefrontal cortex (mPFC), striatum) [18]. The latter process is driven in part by a high attribution of incentive salience to alcohol related cues, whereby over the course of repetitive alcohol use individuals develop an excessive appetitive orientation toward alcohol-related stimuli (i.e., “wanting”) [21, 22]. This increased incentive salience may instigate, increase, or enhance craving and alcohol seeking behaviors [23]. Standard psychotherapy approaches typically engage explicit, top-down regulatory strategies in the service of reducing alcohol use, (e.g., Cognitive-Behavioral Therapy for substance use disorders [24]). Devising interventions that more directly engage bottom-up, automatic processes holds promise for improving clinical efficacy of available interventions targeting both components thought to drive addiction [25, 26].

Cognitive bias modification is an intervention technique designed to adjust maladaptive cognitive-affective biases. In these interventions, participants repeatedly perform one or more tasks designed to reverse or oppose maladaptive biases, typically via computer-guided exercises targeting the automatic processes thought to drive symptoms. One particularly promising form of cognitive bias modification for AUD is Approach Avoidance Training (AAT). AAT for AUD targets approach bias for alcohol-related cues (i.e., automatic action tendencies to approach alcohol-related cues based on implicit appetitive motivational responses). AAT is based on the premise that positively evaluated stimuli typically elicit automatic motor approach behaviors (i.e., approach action tendencies), whereas negative stimuli trigger avoidance [27]. A common task to assess approach and avoidance behavioral responding displays valenced stimuli and asks the participant to pull a joystick (arm flexion; approach) or push it away (arm extension; avoidance) in response to the color of the image border or orientation, with faster approach versus avoidance movements seen for positively evaluated stimuli [28, 29]. Individuals with AUD commonly exhibit a bias demonstrated by faster response to pull versus push alcohol cues [19]. This basic assessment paradigm was adapted to create an AAT *intervention* by establishing a contingency between stimulus valence and required responses to encourage repeated approach or avoidance of cues [30]. In AAT for AUD, participants repeatedly practice behavioral disengagement from alcohol cues (i.e., pushing away), with the goal of changing implicit approach-oriented biases for alcohol related cues.

Growing evidence supports the use of AAT as an adjunctive program with treatment for substance use disorders including AUD [31–36]. A recent randomized clinical trial found that four sessions of AAT increased the likelihood of abstinence by 17% following discharge of inpatient withdrawal facilities [33]; additionally, AAT administered during inpatient treatment has been shown to improve abstinence rates at 1-year follow-up versus control and sham conditions by 8.4% [35]. Facets of approach and avoidance dysfunction have been observed in a variety of psychiatric conditions, suggesting that the presence of comorbid conditions with AUD could impact AAT treatment effects [37–39]. To our knowledge, the efficacy of AAT for AUD has not been evaluated among outpatient participants specifically recruited for presence of comorbid conditions, nor in Veterans. Given the prevalence of AUD (estimated at over 10% [6]) and comorbid conditions in Veterans with AUD, there is a need for further therapeutic development in this population. Finally, the broader impact of AAT on clinical outcomes beyond abstinence remains understudied, which is critical given that abstinence alone is insufficient to capture functional recovery [40–43].

Questions also remain about the underlying neural mechanisms of AAT. Preliminary data suggests that that AAT may shift neural responses in approach system regions during alcohol cue processing. Wiers and colleagues (2015) found that a cognitive bias modification training (AAT) relative to a control condition (Sham) administered to individuals seeking treatment for alcohol use disorder, was associated with a greater reduction in alcohol cue-elicited activation in the bilateral amygdala, which correlated with decreases in craving [44]. A subset of those patients completed an additional scan task assessing behavioral approach tendencies toward alcohol cues (i.e., performed Approach Avoidance Assessment during fMRI), which revealed a significant interaction effect of time by group, where participants who completed AAT showed reductions in the mPFC in the contrast of interest ([alcohol pull > alcohol push] >[soft drink pull > soft drink push]) from pre- to post-training relative to those who completed Sham training [45]. However, to date there have been relatively few studies that have explored neural mechanisms of AAT treatment.

To address these current knowledge gaps, the present study will conduct a randomized controlled trial of AAT versus a comparison condition in Veterans being treated for AUD plus comorbid psychiatric conditions in outpatient Veterans Affairs (VA) care. The trial adopts a mechanistic approach by including comprehensive measurement of outcome across clinical, functional, and neurobiological assessments.

## Methods

### Aims

The primary aim is to determine the efficacy of AAT training compared to Sham training as an adjunctive treatment in Veterans with AUD undergoing standard care. We intend to evaluate if repeatedly practicing avoidance of alcohol cues through AAT cognitive bias modification can improve functional recovery outcomes and reduce hazardous drinking.

The secondary aim is to determine if AAT reduces approach bias for alcohol cues, as measured with behavioral and functional neuroimaging data.

### Trial design

The proposed study is a randomized, double-blind, parallel-group controlled trial. This protocol has been formulated in accordance with the Consolidated Standards of Reporting Trials (CONSORT) 2010 Statement guidelines [46].

### Study setting

Data collection will take place at the VA San Diego Healthcare system. Participants will be recruited through the San Diego VA outpatient addictions treatment program. Upon initiation of treatment, patients who meet study eligibility criteria will be offered the opportunity to participate in the clinical trial in addition to standard care.

### Sample size

Sample size determinations for the functional outcomes were made with a series of power calculations conducted using RMASS power calculation software for longitudinal mixed models. Parameters entered into the calculation included an effect size of d = 0.6 [32], 4 time points, attrition ranges between 20 and 25% based on rates in a prior VA-based clinical trial with a similar sample [47], and α = 0.05. Under the most conservative retention rate, we will need to enroll 136 participants to achieve 80% power. Sample size determinations for imaging outcomes were made using R:WebPower software for analysis of variance for a treatment group x time interaction term. Parameters entered into the calculation included an effect size of d = 0.80, 2 time points, and attrition as above with α = 0.05. We will need a sample of n = 51 to achieve 80% power to detect a treatment group x time effect on the regions encompassed within our a priori mask.

### Participants and study design

**Fig. 1:**
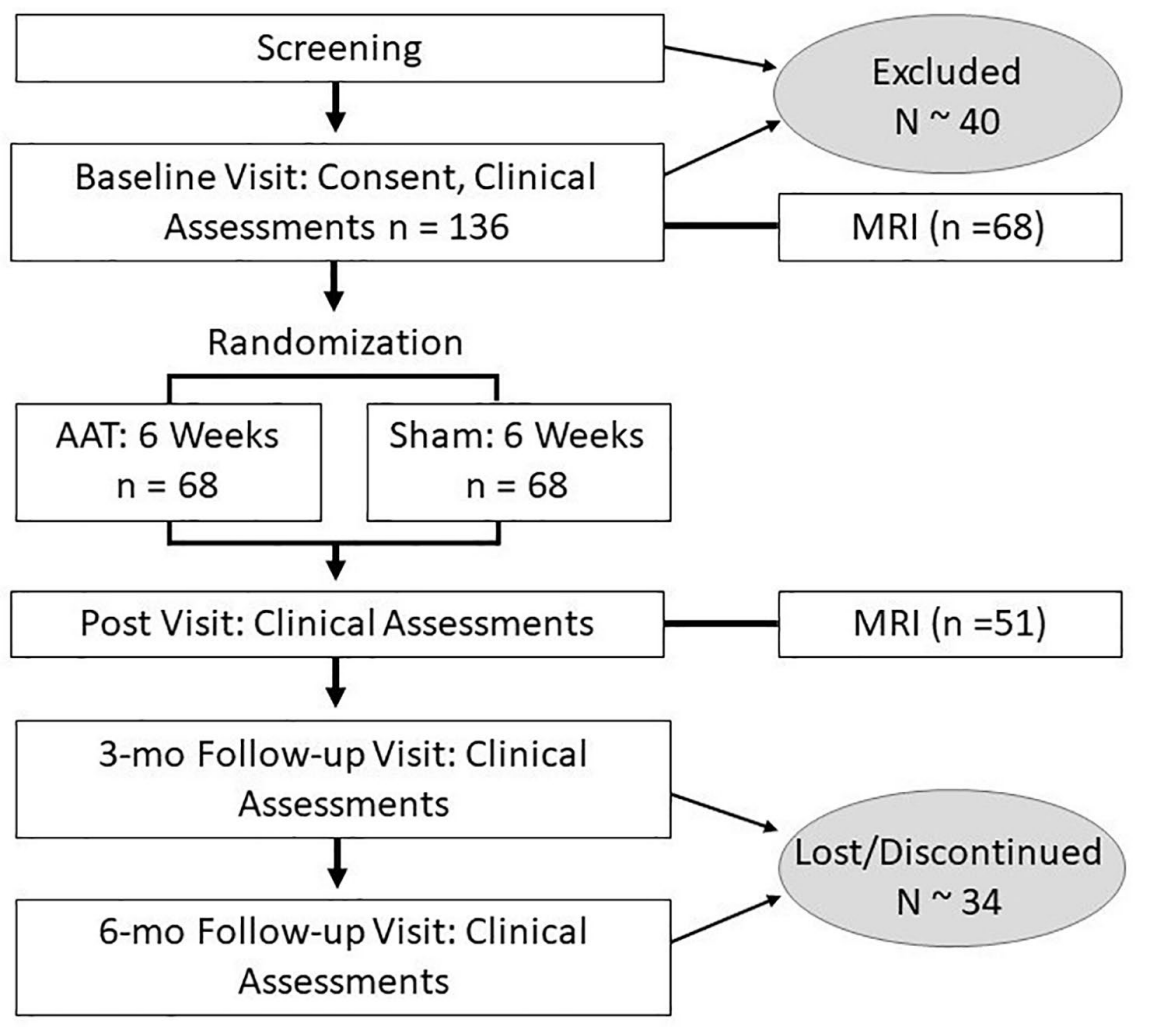
Flow Chart of Study Design and Planned participant enrollment and attrition *Note.* AAT = Approach avoidance training

We plan to evaluate 176 Veterans between the ages of 18–65 with the goal of randomizing 136 participants. Participants will be randomized at the individual level to AAT or Sham. Participants will participate in a functional magnetic resonance imaging (fMRI) in addition to the AAT training and behavioral study assessments, unless they are unable to safely complete scanning procedures (see Fig. 1).

### Eligibility criteria

Participants must be Veterans between 18 and 65 years of age (inclusive of all sexes); have a primary diagnosis of AUD (i.e., meet *Diagnostic and Statistical Manual of Mental Disorders, Fifth Edition* (DSM-5) criteria for AUD); have no more than 90 days abstinence from alcohol prior to study participation; and must have 4-week stability if taking psychotropic medications at the time of enrollment. Participants will be excluded if they have a lifetime history of psychotic or bipolar disorder; neurodegenerative or neurodevelopmental disorders; history of moderate or severe traumatic brain injury or other known neurological conditions; sensory deficits that would preclude completing behavioral tasks; suicidal or homicidal ideation within the past month necessitating urgent higher level care; concurrent individual psychotherapy or other treatment outside of standard clinical programming; conditions unsafe for completing MRI scanning for those completing the scanning component only (e.g., non-scan safe metal in body).

### Measures

#### Eligibility evaluation

At baseline and immediately post-intervention, participants will participate in a structured clinical interview to assess for psychiatric disorders (Mini International Neuropsychiatric Interview for DSM-5), past and current suicidality (Columbia Suicide Severity Rating Scale; C-SSRS), detailed medication and psychotherapy history, MRI safety, and history of head injuries (Ohio State University Traumatic Brain Injury Identification Method Short Form; OSU TBI-ID). Demographic information (e.g., age, biological sex, identified gender, sexual orientation, ethnicity, race, first language, education level, employment, handedness, marital status) will be collected via questionnaires completed by participants at baseline.

### Outcomes

#### AUD-related disability (primary)

The Drinker Inventory of Consequences (DrInC) assesses alcohol-related adverse functional consequences across physical (e.g., harmed physical health), interpersonal (e.g., loss of relationships), role responsibilities (e.g., missed work), psychological (e.g., loss of hobbies), and impulse control-related problems (e.g., legal problems); the first administration establishes lifetime consequences (DrINC-2 L) and a modified version of the DrInC-2R will be used to assess past 30-day consequences, and subsequent administrations assesses past 30-day consequences [48].

#### Recent alcohol and other substance use (secondary)

During each study visit, alcohol consumption will be quantified using the Time Line Follow-Back (TLFB). During this interview, participants are asked to report the number of days alcohol and other drugs were consumed as well as alcohol type,quantity, and beverage size using a calendar method. Percentage days heavy drinking ( > ≡ 4/5 drinks per single occasion for women/men) (PDHD) will be calculated as the TLFB outcome [49]. The TLFB will capture 90 days preceding the baseline appointment. At all subsequent visits, the TLFB will cover the time between interviews.

#### Change in alcohol avoidance behavioral assessment (secondary)

Participants will complete a behavioral Alcohol Approach Avoidance assessment to measure automatic action tendencies to approach alcohol stimuli. Participants will see an alcohol or non-alcoholic beverage picture in the center of the screen framed by either a green or a blue border. They will be instructed to pull the joystick towards themselves when the border is green and to push the joystick away when the border is blue. When the participant pushes the joystick, the picture zooms out and when the participant pulls the joystick the picture zooms in, which creates the visual impression that the pictures are moving away or coming closer, respectively. Participants will be required to make an equal number of approach (pull) and avoidance (push) movements to both alcoholic and non-alcoholic drinks. To control for order and practice effects, there will be two sets alcohol and non-alcohol pictures (sets A and B), matched for beverage category, perceptual features, and national sales data characteristics, that are distinct from the stimuli used during training. Half of the participants in each condition will be presented with picture from set A at pre-test and pictures from set B at post-test (and vice versa). Reaction times are calculated based on the duration from the time the picture appeared on the screen to the time it disappeared. An approach bias score is computed by subtracting each participant’s mean response latency in the pull condition from their mean response latency in the corresponding push condition (e.g., alcohol-push minus alcohol-pull). The assessment block will comprise 128 trials: 16 Pictures x 2 Picture Type (alcohol vs. non-alcohol) x 2 Border Color (green vs. blue). Alcohol pictures include images of beer, wine, spirits, and mixed alcoholic beverages, each presented an equal number of times. The task outcome is change from baseline to post-intervention in behavioral approach bias score between baseline.

#### Change in alcohol approach avoidance imaging assessment (secondary)

While undergoing fMRI, participants will complete a modified Alcohol Approach Avoidance assessment. In this task, 20 alcohol images and 20 neutral images matched for perceptual features are presented in a pseudo-randomized order (i.e., streaks greater than 4 are eliminated), with a total of 160 trials over 2 blocks. Images are distinct from those used in the behavioral and training tasks. As the goal is to assess the neural correlates of approach bias, the pictures will be pushed and pulled equally often (50% alcohol / 50% neutral). Pushing and pulling cues are accompanied by the visual feedback of the cue zooming out and in, respectively. Participants are told to “make the picture disappear as soon as possible,” and to pull or push in response to a green or blue border, respectively. They have 2.5 s to respond to a picture before auto-forwarding and the trial discounted. Intertrial intervals are 0.5–10 s distributed at random (jittered). The task outcome is blood oxygen level dependent (BOLD) response to a primary contrast comparing alcohol to neutral beverage for push versus pull (alcohol pull – alcohol push > neutral pull – neutral push).

### Intervention

Veterans will be randomized to an AAT or Sham Condition. In these paradigms, participants are first shown a fixation cross, followed by an image (alcohol or neutral beverage) with a colored border surrounding it. They then use a joystick to respond to the color of the border surrounding the stimulus image (i.e., pull for green, push for blue). To experimentally manipulate automatic action tendencies, in AAT a contingency is set between alcohol stimuli (i.e., alcoholic beverage images) and avoidance behaviors (i.e., arm extension or push) in the active AAT condition. Thus, in the active condition, the majority of alcohol images (92%) are presented with a blue border that indicates an instruction to push (i.e., avoid). Participants randomized to the Sham condition will complete a computer-delivered program that is similar to AAT, and matched for number of sessions, but without a contingency between instruction type and pictures (i.e., 50% pull). The AAT and Sham paradigms consist of 192 trials, including 16 alcohol images (4 of each image type: beer, wine, spirits, and mixed beverages) and 16 neutral images, where each image repeats 6 times. Participants will be instructed to complete the AAT or Sham tasks twice weekly, starting from their first week in outpatient treatment, for six weeks. The programs will be completed using a joystick either in an exam room on site or remotely. Participants choosing to complete the intervention remotely will be provided a joystick.

### Monitoring

A data monitor will oversee the present study. The main responsibilities of the data monitor will include reviewing the protocol prior to recruiting, meeting annually with the research team, and annual monitoring of study activities and oversight of any adverse events for safety purposes. The local San Diego Healthcare System Institutional Review Board will conduct an annual audit of the present study which includes a review of enrollment, consent, eligibility, adherence to trial procedures and policies to protect participant confidentiality, and reporting of adverse events.

The safety monitor will oversee the study and report any issues regarding safety or threats to data integrity to the IRB. In the present study, all Serious Adverse Events (SAEs) (i.e., any fatal event, immediately life-threatening event, permanently or substantially disabling event, or event requiring or prolonging inpatient hospitalization) will require immediate review and will be reported to the safety monitor regardless of any judgement of their relatedness to the study. SAEs will be reported to the local IRB within 24 hours of the SAE. A summary of Non-Serious Adverse Events will be provided to the safety monitor annually. Additionally, a summary of participant retention and reasons for participant dropout by experimental condition will be provided (by KH) to the safety monitor.

### Allocation

Randomization will occur during the second visit after eligibility criteria have been confirmed. Randomization sequences will be generated by the study quantitative psychologist, who is not involved in recruitment or data collection, using a computer-generated randomization sequence, using R (*Base* package*)* [50] and administered through RedCAPs randomization function. Randomization will occur without the ability to predict a participant’s allocation before randomizing. Randomization will be blocked (block size = 4 participants; 34 blocks total) and stratified by age category (2 strata: < 40 vs. > = 40 years old) given that age is known predictor of AAT response [31]. The study design will be double-blind, i.e., the participant, assessor, and PIs will be masked to the treatment condition for the duration of the trial with no planned unblinding. Within each stratum, participants will be divided into four groups, i.e., 2 treatment conditions (AAT vs. Sham) X 2 image set orders for Pre and Post Assessment AAT (AB vs. BA), with an allocation ratio of 1:1:1:1. Each computer program corresponding to each of the 4 conditions will be blinded such that the coordinator will not know the nature of the program. Assessors will not deliver the intervention. The patient and assessor will each rate their prediction regarding the patient’s assigned treatment condition after each assessment to check the success of blinding.

### Procedure

Data collection is planned to take place between February 2023 and May 2026. Eligible participants will be enrolled in concert with clinical treatment initiation. Following a brief telephone screening, participants will complete a baseline visit that includes written informed consent, structured clinical interviews, and self-report questionnaires. At the time of consent, participants will be informed that they may refuse to participate or discontinue at any time and any participants wishing to withdraw will be given referrals for VA mental health services. If a participant expresses significant distress or discomfort during any procedures, the principal investigator will be notified and provide appropriate referrals or other clinical action. If eligibility is confirmed, participants will complete a second visit that includes the behavioral task (Alcohol Approach Avoidance Assessment). If participants are eligible to undergo fMRI scanning, they will complete the modified Alcohol Approach Avoidance Assessment while in the scanner. Training will be scheduled upon completion of visit 2. Participants will be scheduled to complete visits twice weekly for six weeks but will have up to 10 weeks to complete the 12 training sessions in the event of rescheduling. Immediately following the final training session, participants will complete two post treatment visits that mirror the first two assessment visits. Three months and six months after baseline, a research team member will administer the TLFB and assess treatment change. At these final two treatment follow-up appointments, participants will complete self-report questionnaires. See Table 1 for an outline of measures included in each appointment.

**Table 1:** Measures by Visit

	Enrollment		Post-allocation			
Timepoint	Screening		Visit 2	Post	3-mo	6-mo
**Screening**:						
Demographics		X				
MINI		X		X		
C-SSRS		X		X		
OSU TBI-ID		X		X		
Medical History		X		X		
Treatment History		X		X		
MRI Safety		X		X		
**Recovery Outcomes**:						
DrInC		X		X	X	X
TLFB		X		X	X	X
**Approach Bias (Approach Avoidance Assessment)**:						
Behavioral			X	X		
fMRI			X	X		

*Note*. Columbia Suicide Severity Rating Scale = C-SSRS; DrInC = Drinker Inventory of Consequences; MINI = Mini International Neuropsychiatric Interview for DSM-5; Ohio State University Traumatic Brain Injury Identification Method Short Form = OSU TBI-ID; Timeline Follow-Back = TLFB

## Outcomes and statistical methods

### Primary and secondary clinical outcomes

To evaluate whether AAT significantly improves clinical outcomes relative to Sham, a mixed effects model repeated measures (MMRM) model will be used. The dependent variable in the MMRM model is change from baseline in AUD-related disability (DrInC) at each post-baseline visit (post visit, 3 and 6 mo. follow-up). Predictors in the model will include treatment condition (AAT, Sham), visit (immediately post-training, 3 and 6 mo.), condition by visit interaction term, and baseline DrInC. The primary end point is the post-training visit. The same analytic approach will be applied to the secondary outcome (PDHD). We will model the distribution of percent days heavy drinking to ensure appropriate analysis. All subjects randomized will be included in the intent-to-treat analysis. Depending on the type of missing data, the appropriate statistical approach (e.g., multiple imputation) will be determined. Sensitivity analyses will be used to explore the impact of missing data.

### Secondary behavioral outcomes

To assess change in alcohol-related approach bias and cognitive performance, we will use the Approach Avoidance Assessment approach bias score (reaction time in seconds; alcohol-push minus alcohol-pull). To determine whether AAT engages the target as hypothesized (i.e., improving approach bias), we will analyze the effect of AAT versus the Sham group in a mixed effects model repeated measure approach as in Aim 1.

### Secondary MRI outcomes

#### Single subject analysis

Imaging data will be collected on Siemens 3T Prisma scanner. The Alcohol Approach Avoidance Assessment task will be scanned with echo-planar imaging (EPI) sequences and a high-resolution anatomical image will also be collected. Data preprocessing will occur with our standard pipelines with AFNI and ANTsR software [51, 52]. To assess approach bias, individual task regressors will be generated for each participant to account for individual differences in response and movement speed. Task activation maps for push conditions will then be subtracted from the pull conditions to create the primary contrast of interest [alcohol pull-push > neutral pull-push].

#### Neuroimaging group level analysis

Linear mixed effects models will be used to evaluate whether BOLD activation changes differentially over time and group (AAT training versus Sham) for the primary contrast of interest. The primary fMRI outcome is the difference in BOLD response to the alcohol pull-push > neutral pull-push contrast, which is a continuous outcome variable reflecting differential activation to the task conditions at each voxel of the brain. We will use a threshold adjustment based on Monte-Carlo simulations (AFNI’s 3dClustSim) to ensure Type I error is controlled when interpreting significant brain clusters that are active. The simulations will be generated in a single mask of three a priori anatomical regions of interest (ROIs) previously identified as impacted by AAT in the context of an RCT to treat AUD, including the bilateral amygdala, mPFC, and striatum. The linear mixed effects model conducted in AFNI 3dLME will include condition (AAT vs. Sham) as a between-subject factor and time (baseline, post-training) as the within-subjects factor, with participant included as a random factor. The hypothesis that AAT recipients, relative to Sham recipients, will demonstrate reduced approach bias would be supported by an interaction of condition by time on BOLD activation in the primary contrast of interest.

### Data management and dissemination

All data entry and management will be conducted in accordance with requirements by the VA San Diego Health Care System and the Standards for Privacy of Individually Identifiable Health Information (“Privacy Rule”) of the Health Insurance Portability Act of 1996 (“HIPAA”). To increase confidentiality, all data will be de-identified prior to data entry into secure databases. To ensure data quality, data will be double entered. Analysis and publication of the study results will be conducted by the investigative team without the involvement of external professional writers. All personnel who have had a significant role in development, analysis, or writing will have the right to authorship of the manuscript reporting the primary outcomes. JB and AST will determine authorship of manuscripts reporting additional secondary analyses based on the individuals’ role to the aspects of the study being reported.

## Discussion

This study protocol described a double-blind randomized controlled trial to assess the clinical utility of AAT as an adjunctive treatment to standard care in Veterans with AUD and comorbid psychiatric disorders. Prior research supports the use of AAT as an adjunctive treatment for substance use disorders [34, 44, 53–55] and emerging research suggests that variations of cognitive training for approach/avoidance biases may be beneficial for symptoms of other psychiatric disorders [29, 56, 57]. However, the impact of AAT treatment on alcohol consumption and functional recovery outcomes in Veterans with AUD and comorbid psychiatric disorders remains unaddressed. The present study protocol aims to address this gap by including comprehensive assessments of functional recovery in addition to investigating if AAT reduces approach bias for alcohol cues as measured at the behavioral and neural level.

One strength of the study design is the comprehensive evaluation of changes in approach bias across behavioral and neural indicators. Previous research suggests AAT may modify neural regions implicated in alcohol addiction and relapse (e.g., mesolimbic regions) [45], resulting in greater reductions in cue-elicited activation in the bilateral amygdala and decreased craving in alcohol dependent individuals [44]. Assessing changes in brain response to AAT may provide evidence to validate the proposed model suggesting that aberrant approach bias, driven by incentive salience dysfunction, is a modifiable factor underlying pathological alcohol use.

It is important to address potential limitations and possible mitigation approaches associated with conducting the proposed study. The main challenges include treatment adherence, participant retention, and study completion since dropout rates for addiction treatments are high, especially in comorbid populations [58, 59]. As attrition rates are anticipated to range between 20 and 25%, participants will be overrecruited to ensure the study is well powered to examine treatment effects. The treatment protocol includes 12 sessions, administered twice a week for 6 weeks, which can be demanding for participants who are also receiving concurrent clinical care. AAT treatment sessions will therefore be made as convenient as possible by allowing in-person or remote appointments, depending on participant preference, in order to reduce participant burden. Procedures will also be put in place to remind participants of their appointments and provide opportunities to easily reschedule missed treatment sessions. To support retention, participants are asked to provide mobile phone numbers, home addresses, and a secondary phone contact to ensure treatment reminders are received in a timely manner. Participants are compensated for study participation after completion of each follow-up appointment. Task-based adherence data will be collected during the computerized treatment sessions to assess if participants are engaging sufficiently in treatment sessions.

In conclusion, the current study will test AAT with standard care in Veterans given evidence that this may be a promising adjunctive treatment for individuals with AUD and comorbid psychiatric disorders. If found effective, AAT could serve as a non-invasive, low cost, mechanistic specific treatment targeting approach bias by using a cognitive training procedure designed to reduce approach bias toward alcohol cues. This new treatment approach has the potential to reduce healthcare costs by improving recovery outcomes for Veterans and consequently reducing treatment readmittance. In addition, offering AAT as an adjunctive treatment to standard care presents a relatively low financial burden with high potential for successful outcomes. Research findings are anticipated to contribute valuable data to the field by demonstrating links between approach bias shifts and clinical outcomes with an interdisciplinary study design. This trial will further our understanding of AAT treatment mechanisms and provide valuable clinical data on AUD-related functional outcomes in Veterans with comorbidities.

## Electronic supplementary material

Below is the link to the electronic supplementary material.

Supplementary Material 1

Supplementary Material 2

## Data Availability

The datasets generated during the current study will not be publicly available but are available from the corresponding author on reasonable request in consideration of VA data management and access plan policies. For further information about the protocol, all items from the World Health Organization Trial Registration Data Set are detailed in the appendix table.
