# Supplementary material for: Approach avoidance training versus Sham in veterans with alcohol use disorder: protocol for a randomized controlled trial
Source: BMC Psychiatry. 2023 Jul 12;23:499. doi: 10.1186/s12888-023-04961-z (PMC10337098; doi:10.1186/s12888-023-04961-z)
Supplement: Supplementary file 1 — Supplementary Material 1 [file 12888_2023_4961_MOESM1_ESM.docx]

**Appendix A**

**Table A1**

*World Health Organization Trial Registration Data Set.*

| **Data category** | **Information** |
| --- | --- |
| Primary registry and trial identifying number | ClinicalTrials.gov: NCT05372029 |
| Date of registration in primary registry | 5/9/2022 |
| Secondary identifying numbers | N/A |
| Source(s) of monetary or material support | Department of Veterans Affairs and by the VA Center of Excellence in Stress and Mental Health |
| Primary sponsor | VA Office of Research and Development  Contact: Patricia A Franklin, AA BS  858-552-8585 ext 7441 [patricia.franklin2@va.gov](mailto:patricia.franklin2%40va.gov?subject=NCT05372029,%20D3793-R,%20AAT%20for%20Alcohol%20Use%20Disorder%20in%20Veterans) |
| Secondary sponsor(s) | N/A |
| Contact for public queries | Jessica Bomyea, PhD, Phone: 858.822.4120; Email: jbomyea@health.ucsd.edu |
| Contact for scientific queries | Jessica Bomyea, PhD, Department of Psychiatry, University of California, San Diego, La Jolla, CA 92037 |
| Public title | AAT for Alcohol Use Disorder in Veterans |
| Scientific title | Enhancing Treatment Outcomes Among Veterans With Alcohol Use Disorder: Clinical and Neural Markers of Adjunctive Approach-avoidance Training |
| Countries of recruitment | United States |
| Health condition(s) or problem(s) studied | Alcohol Use Disorder |
| Intervention(s) | Behavioral: Approach Avoidance Training  Behavioral: Sham Training |
| Key inclusion and exclusion criteria | Inclusion Criteria: fluent in English, primary diagnosis of AUD with no more than 90 days abstinence from alcohol, 4-week stability if taking psychotropic medications  Exclusion Criteria: lifetime history of psychotic or bipolar disorder, neurodegenerative or neurodevelopmental disorders, history of moderate or severe traumatic brain injury or other known neurological condition, sensory deficits that would preclude completing tasks, suicidal or homicidal ideation within the past month necessitating urgent higher level care, concurrent individual psychotherapy or other treatment outside of standard DDRP programming, conditions unsafe for completing MRI scanning for those completing the scanning component only (e.g., metal in body) |
| Study type | Interventional (Clinical Trial)  Allocation: randomized  Intervention model: parallel assignment  Masking: double blind (Participant, Outcome Assessor  Phase: N/A  Primary Purpose: Treatment |
| Date of first enrolment | February 2023 |
| Target sample size | 176 participants |
| Recruitment status | Recruiting |
| Primary outcome(s) | Change in the Drinker Inventory of Consequences (DrInC) |
| Key secondary outcomes | 1. Change in the Timeline Follow-back Procedure (TLFB)  2. Change in Alcohol Approach Avoidance Behavioral Assessment (approach bias score)  3. Change in Alcohol Approach Avoidance Imaging Assessment (BOLD neural signal) |

**Appendix B**

**Sample Patient Informed Consent, HIPPA, and Exprimental Subject’s Bill of Rights**

|  | **Agreement to Participate in Human Subject Research**  IRB Protocol #: **H220043** |
| --- | --- |
| **Study Title:** *Enhancing Treatment Outcomes Among Veterans With Alcohol Use Disorder: Clinical and Neural Markers of Adjunctive Approach-Avoidance Training* | |
| **Principal Investigator:** Jessica Bomyea, Ph.D. | |
| **VA Facility:**  VA San Diego Healthcare System | |

Participant Name: Date:

| **STUDY SUMMARY** |
| --- |

You are being asked to participate in a research study. This section summarizes key information about this study to assist you, or your legally authorized representative, in understanding the reasons why you may or may not want to participate in the research. Your participation is voluntary. You may refuse to participate or withdraw at any time. You will not lose any services, benefits or rights you would normally have if you choose not to volunteer. Carefully review this section and the detailed information that follows before you agree to participate.

***WHAT IS THE STUDY ABOUT AND WHY ARE WE DOING IT?***

This study is about examining the effects of a computer-based cognitive training treatment program on symptoms of alcohol use disorder (AUD). It is being funded by the Department of Veterans Affairs. By doing this study, we hope to learn about the usefulness of the program, in conjunction with standard care, for helping Veterans reduce their hazardous drinking and recover from alcohol-related problems in day-to-day life.

***what does the study involve AND HOW LONG WILL IT LAST?***

You will be asked to complete asked a series of questions about your mental health and drug and alcohol use history using questionnaires and interview questions. You will be asked to participate in several computer tasks that measure your reaction times and accuracy. You may be asked to complete a magnetic resonance imaging (MRI) visit. You will then be randomly assigned to one of two groups. Regardless of group assignment, you will be asked to participate in twelve computer-based cognitive training sessions that involve responding on a computer to images of alcohol and/or neutral beverages. Your participation in this research will last about six months. The total duration of the study is approximately 5 years.

***what are key reasons you might choose to volunteer for this study?***

You may experience a decrease in symptoms related to AUD as a result of treatment during your participation. The results of this study will provide information to help inform new treatments for individuals experiencing symptoms of AUD. We cannot guarantee, however, that you will receive any benefits from participating in this study.

***what are key reasons you might choose not to volunteer for this study?***

You might not want to participate in this study because there are potential risks of discomfort including fatigue, discomfort while being asked personal questions, and certain risks of doing MRI scans which we describe further below. The study also requires additional time and activities beyond your standard care. A complete description of risks is included in the Research Details Study Risks section. There are alternative treatments for AUD. A complete description of alternate treatment/procedures is provided in the Research Details Alternatives section.

***what if you have questions, suggestions or concerns?***

The people in charge of the study are Jessica Bomyea and Andrea Spadoni Townsend of the VA San Diego Healthcare System. If you have questions, suggestions, or concerns regarding this study or you want to withdraw from the study their contact information is: 858.552.8585x1416.

| **RESEARCH DETAILS** |
| --- |

***WHO IS CONDUCTING THIS RESEARCH AND WHY?***

Drs. Jessica Bomyea and Andrea Spadoni Townsend are asking for your consent to this research. This study is being sponsored by the Department of Veterans Affairs. No members of the team have any significant financial interest of other conflicts of interest related to the research project.

The purpose of the research is to evaluate the usefulness of the program, in conjunction with standard care, for helping Veterans reduce their hazardous drinking and recover from alcohol-related problems in day-to-day life. Outcomes of a computer-delivered cognitive training program will be compared to those of a similar computer-delivered program that is believed to have little to no beneficial effect on symptoms. The research is being done to test whether this cognitive training program may be a helpful addition to treatment for Veterans with AUD. We are also examining how this kind of treatment may impact brain functioning using MRI. You have been asked to participate because you (1) are a Veteran who meets DSM-5 criteria for AUD, (2) are between the ages of 18-65, (3) are willing to attend assessment and treatment sessions (4) and are currently enrolled in the San Diego VA Dual Diagnosis Recovery Program (DDRP) treatment program. Approximately 136 people will take part in this research at this facility.

***FOR HOW LONG WILL I BE IN THE STUDY?***

Your individual participation will require approximately 18 visits over a six-month period. There will be two visits, one immediately before treatment and one immediately after treatment, that will last an average of 2.5 hours each. There will be visits at 3 months and 6 months after treatment will last approximately 1 hour each. There will be 12 visits for the purpose of completing the computerized cognitive training program only and will last approximately 20-30 minutes each. If you are asked to participate in MRI scanning visits, there will be two visits that will last approximately 2 hours each. The total time required for participation if you are asked to complete the scan session is approximately 17 hours, if not it will take 13 hours.

***WHAT WILL HAPPEN AND WHAT CAN I EXPECT IF I TAKE PART IN THIS STUDY?***

If you agree to be in the study, the following will happen to you:

1. You will be asked to answer a series of questions about your mental health and alcohol use history by an interviewer in order to determine if you are eligible to participate in the remainder of this study. If you are not eligible to participate in the remaining parts of the study, the information obtained from you in the interview will be omitted from this study and destroyed to protect your privacy. If your responses indicate that you *are* eligible to complete the remaining parts of this study, you will be asked to continue.

2. You will be asked to answer a series of questionnaires about your mental health history, how you feel, and experiences that you have had. You can skip any question that makes them uncomfortable and can stop at any time.

3. You will be asked to participate in several different computer-based tasks that measure your reaction time and response accuracy. These computer tasks will take approximately 30 minutes. You may take breaks between the computer tasks.

4. You may be asked to participate in a brain imaging component of the study before and after completing treatment. If so, a picture of your brain will be obtained using an MRI scanner. During the MRI scan, you will be placed in a large donut-like machine. Your head will be placed in a helmet-like holder that allows us to take images of your brain. During the scanning session, you will be asked to complete computerized tasks. These tasks will include doing one or more of the following: (1) solving problems, (2) making decisions based on personal preferences, (3) remembering pieces of information for a short period of time, or (4) looking at alcohol-related images.

At the time of your first visit, the research team will determine if you are appropriate for the MRI based on the absence of the following conditions: cardiac pacemaker, metal fragments in eye, skin, body; heart valve replacement, brain clips, venous umbrella, being a sheetmetal worker or welder, aneurysm surgery, intracranial bypass, renal, aortic clips; prosthetic devices such as middle ear, eye, joint, or penile implants, joint replacements; hearing aid, neurostimulator, insulin pump; I.U.D; being pregnant or trying to become pregnant; shunts/stents, metal mesh/coil implants; metal plate/pin/screws/wires, or any other metal implants; permanent eyeliner, eyebrows, some tattoos; or any other conditions that would make it unsafe to scan you in the MRI (including concerns regarding the risk of COVID19 transmission). If you are found to have any of the aforementioned objects or conditions, you will not be scanned but you may continue with other portions of the study. You may also opt not to do the MRI if you wish, even if you are eligible to do so.

If you are asked to do an MRI scan, you will be administered a breathalyzer directly prior to your MRI scan. This is administered in order to ensure your safety while in the MRI scan. If the breathalyzer indicates recent alcohol consumption, we will ask to reschedule your appointment to a different day

If you are asked to do an MRI scan and are a female and capable of child-bearing, a urine-based pregnancy test will be administered prior to your MRI scan in order to be as sure as possible that you are not pregnant. It is important to be as sure as possible that you are not pregnant, because it is currently unknown whether or not exposure to the magnetic fields from an MRI scanner is a risk to a fetus. Only those women who have a negative pregnancy test result may participate in the study. Our research team will provide you with a pregnancy test to self-administer prior to the MRI scan.

5. You will be randomly assigned to one of two groups. You will be put into a study group by chance (like a coin toss), with an equal probability of assignment to each group. The group you are assigned to will determine which type of computer-based program you will complete. Group One will receive the computer-based program that is designed as a cognitive training intervention, and Group Two will receive the computer-based non-training (placebo). You and individuals conducting assessments for the study will not know which group you have been assigned to until the end of the study. You cannot choose your study group.

After you have been randomly assigned to a group, you will be asked to participate in twelve computer-based training visits. These visits will occur twice per week over a period of six weeks and will last approximately 20-30 minutes each. During these visits, you will be asked to respond to alcohol-related images and neutral beverage images. Completing these tasks does not require any computer skills. These computerized training sessions are only available to individuals actively enrolled in the study and are not available after the conclusion of study participation.

6. Information from your medical record may be utilized as noted in the HIPAA Authorization.

The study will take place at the San Diego VA Healthcare facilities, with the exception of these MRI scans that occur at the UCSD Keck Center for Functional MRI, which is approximately ½ mile from the VA hospital. You may be offered the option of completing assessments and computerized training via the telephone and using remote telehealth technologies, rather than completing visits in-person, if it is deemed to be in your best interest to do so (e.g., to satisfy social distancing requirements due to COVID-19 concerns).

Responsibilities and expectations as a participant in this study:

- Complete the treatment sessions as instructed.
- Keep your study appointments. If you miss an appointment, please contact the investigator or research staff to reschedule as soon as you know you will miss the appointment.
- Alert study staff to any changes in your DDRP or other mental health treatment.
- Tell the investigator or research staff if you believe you might be pregnant.
- Complete your questionnaires as instructed.
- Ask questions as you think of them.

Sometimes over the course of a research study, research personnel may encounter incidental findings. An incidental finding is a previously undiagnosed medical or psychiatric condition that is discovered unintentionally. Research personnel are not trained to diagnose potential abnormalities. In such a case, a qualified medical professional will be consulted. If this condition requires further medical evaluation or treatment, you will be notified.

***WHICH PROCEDURE/S OR TREATMENT/S ARE DONE FOR RESEARCH?***

Treatment that you receive in the DDRP clinic is standard of care. The research procedures outlined above (including assessments, MRI scanning, and computer-delivered cognitive training treatment) are for research purposes.

***WHAT POSSIBLE RISKS OR DISCOMFORTS MIGHT I HAVE IF I TAKE PART IN THIS STUDY?***

Any procedure has possible risks and discomforts. The procedures in this study may cause all, some, or none of the risks or side effects listed. Rare, unknown, or unexpected risks also may occur.

Risks associated with assessment tasks:

Some people become uncomfortable at being asked questions about their mental health. If, for any reason, you wish not to answer specific questions or you wish to terminate the session, you will be able to do so.

A. Common risks

- restlessness, anxiety, or fatigue in some people while filling out questionnaires.

- temporary discomfort while being asked personal questions about medical history.

- temporary discomfort or frustration while being asked to complete challenging computer tasks that ask you to use thinking skills (e.g., memorizing) or view emotional cues.

B. Occasional

- muscle aches due to lying on your back for 1 hour in the scanner.

- discomfort due to banging noises that the MRI scanner makes while taking pictures. You will be asked to wear earplugs to minimize the risks of these loud noises to your hearing.

- muscle twitches during the scanning procedure.

C. Rare

- damage to your personal reputation, ability to become or remain employed, or exposure to criminal or civil liabilities, or other unforeseen consequences, if your confidential information were to accidentally become public- e.g., whether you have used illegal substances. To minimize this risk, all of your data will be anonymized and kept in locked cabinets or in databases with secured passwords.

There is always a chance that any procedure can harm you. In addition to the risks described above, you may experience a previously unknown risk or side effect.

Risks associated with MRI:

Some people experience a 'closed-in' feeling due to the relatively restricted space within the MRI machine. You may not be able to have the MRI procedure if you have certain metal, surgical clips, or implants, including a brain aneurysm clip or a pacemaker, in your body, because during the MRI procedure metal can heat up and move, or clips and implants may stop working. Dental fillings are not a problem. If there is any question about whether or not there is metal in your body, you may be requested to have an X-ray to determine this; the X-ray will become part of your medical record. If you are pregnant, you should delay having an MR scan. Women of child-bearing potential will be asked to take a pregnancy test. You will need to remove all jewelry or clothing with metal before having the MRI. All of these precautions will be reviewed with you immediately before you have the MRI. You may experience a brief sensation of vertigo when moving through the scanner. Keep in mind that you may stop the scan at any time by pressing a button that will immediately notify investigators that you would like to exit.

Photographs, audiotaping, or videotaping:

The study team has explained that by signing this Informed Consent Document under the section “I would like to participate in having portions of my assessments audio recorded” below, you voluntarily and without separate compensation authorize voice recording(s) to be made of you by the research team while you are participating in this study. Voice recording is intended for the following purposes: evaluation of reliability of the assessor (i.e., to check that all participants are asked the questions in the same way). The study team has also explained that you will not receive any royalty, fee or other compensation for such use. If you refuse to grant consent, there will be no effect on any VA benefits to which you may be entitled. You may at any time exercise the right to cease being recorded, and may rescind your consent for up to a reasonable time before the voice recording is used.

I would like to participate in having portions of my assessments audio recorded:

Signature: ________________________________ Date: ________________

If you do not wish to be recorded, please do not sign the line above.

***WHAT ARE THE POSSIBLE BENEFITS OF THIS STUDY?***

We do not know if you will get any benefits from taking part in this research study. However, possible benefits may include a reduction in AUD-related symptoms or improvement in perceived recovery during your participation. The information we get from this study might help others with your conditions.

***WHAT OTHER CHOICES DO I HAVE IF I DO NOT WANT TO JOIN THIS RESEARCH STUDY?***

There are alternative treatments for AUD. These include cognitive-behavioral and talk therapy, as well as medications that have been shown to reduce addiction cravings and relapse. You may discuss these options with your doctor.

**WILL I BE TOLD NEW INFORMATION ABOUT THIS STUDY?**

While you are a participant in this study, you will be notified if any important new information is found that may affect your willingness to continue. If the results of this research might influence your medical care after you complete participation, the investigators will contact you to let you know these results.

***WHAT WILL HAPPEN IF I AM INJURED BECAUSE OF MY BEING IN THE STUDY?***

The VA will provide necessary medical treatment should you be injured as a result of participating in this study and following study procedures. You will be treated for the injury by the VA at no cost to you or your insurance but no additional compensation is available. If you should have a medical concern or get hurt or sick as a result of taking part in this study, call Dr. Jessica Bomyea or Andrea Spadoni Townsend at 858.552.8585 x1416.

***DO I HAVE TO TAKE PART IN THIS STUDY?***

Taking part in this research study is your decision. Your participation in this study is voluntary. You do not have to take part in this study, but if you do, you can stop at any time. You have the right to choose not to participate in any study activity or completely withdraw from continued participation at any point in this study without penalty or jeopardy to the medical care you will receive at this institution or loss of benefits to which you are entitled. For data already collected prior to your withdrawal, the investigative team may continue to review data already collected for the study but cannot collect further information, except from public records.

***RIGHT OF INVESTIGATOR TO TERMINATE MY PARTICIPATION***

The investigators may terminate your participation in the study if they believe it is in your best interest, including if your treatment team determines that you would benefit from a different level of care.

***WHAT ARE THE COSTS TO ME IF I TAKE PART IN THIS STUDY?***

There will be no costs to you or your insurance for any procedures or testing done only as part of this research study. If you receive a bill for services that you think could be related to your participation in this study, you should contact Dr. Bomyea or Spadoni Townsend. Medical care and services provided by the VA that are not part of this study (e.g., normal hospital and prescription expenses which are not part of the research study) may require co-payments if your VA-eligibility category requires co-payment for VA services.

***WHAT COMPENSATION WILL I RECEIVE IF I TAKE PART IN THIS STUDY?***

For completing the first round of assessments (prior to treatment), you will receive $20. For completing the second round of assessments (after treatment), you will receive $20. For completing the third round of assessments (3 months after treatment), you will receive $20. For completing the fourth round of assessments (6 months after treatment), you will receive $20. For completing all of the training sessions, you will receive $60. If you complete all study procedures you will receive a $20 completion bonus. If you are asked to complete the MRI component of the study, you will receive $100 for each scan (one before treatment, and one after treatment). You will receive $10 if you arrive for your first visit and it is determined that you are not eligible to participate in the remainder of the study. The most common reason for this determination is that information comes to light which renders it unsafe for you to continue. In total, if you complete all study procedures except MRI sessions you will be compensated $160. If you complete all study procedures including the MRI you will be compensated $360. Subjects can also request an artistic digital non-PHI image of their brain which we will send over VA-approved encrypted software.

In addition, travel reimbursement will be provided to subjects who are unable to participate because of travel expense incurred. Travel reimbursement will be provided at the VA listed reimbursement rate (https://www.va.gov/health-care/get-reimbursed-for-travel-pay/#reimbursedexpenses-and-rates). This website will be referred to every time travel reimbursement is required to ensure participants are being reimbursed at the proper rate.

Each payment will be made directly to your bank account using electronic funds transfer. Initiation of payment after each visit will be completed within approximately 3 business days, but payments are disbursed centrally by the Financial Management System and processing times vary. If you currently have a debt to the Federal Government, your debt may be subtracted from your funds transfer payment for study participation. Payments are processed using Internal Revenue Service Form 1099 which requires the use of your Social Security Number (SSN).

**WHO DO I CONTACT ABOUT THIS STUDY IF I HAVE QUESTIONS?**

If you have any questions, complaints, or concerns about the research or other related matters, you may contact Dr. Bomyea or Spadoni Townsend at 858-552-8585x1416 or the Research Team at x2872. If you have any questions or concerns about your rights as a research subject, the validity of a research study, or research personnel you can contact the Research Compliance Officer at 858-642-3817, VA Research Service at 858-642-3657, VA Regional Counsel at 858-642-1540, or the VASDHS Institutional Review Board at 858-642-6362. This is the Board that is responsible for overseeing the safety of human participants in this study.

***FUTURE USE OF DATA AND RE-CONTACT***

If you are interested in receiving information regarding additional research opportunities, you may be contacted by phone or mail and given information about other studies by a member of our VA investigative team. Whether or not you choose to participate in these studies is entirely your choice. Participation in other research studies will not affect your ability to continue in the current study. Please initial one of the boxes below to indicate your decision:

**Yes, I may be contacted for future research opportunities as described**. _________ (initial)

**No, I do not wish to be contacted for future research opportunities as described**. _________ (initial)

***HOW WILL MY PRIVATE INFORMATION BE PROTECTED?***

Participation in this study may involve a loss of privacy, but information about you will be handled as confidentially as possible. We will include information about your study participation in your medical record. We will collect your SSN which is required for payment as described above.

We will keep confidential all research and medical records that identify you to the extent allowed by law. Your research records will contain no names and will be labeled with a code number. The list that matches your name to the code number will be kept electronically behind a firewall-secured, password protected file. Paper research records will be kept in a secure VASDHS location, or as digital files behind the secure VASDHS computer firewall. None of the presentations or publications based on the data collected through this study will identify you. To minimize risk of confidentiality loss during procedures delivered remotely (e.g., phone assessments), you will be asked to complete all activities in a private environment where others cannot see or hear you. However, you should know that there are some circumstances in which we may have to show your information to other people. For example, the Federal Office of Human Research Protection, the General Accounting Office, the VASDHS R&D Committee, the VASDHS Institutional Review Board, the Food and Drug Administration, and federal compliance officers may look at records that identify you. The UCSD MRI Center may be able to view de-identified MRI scans of your brain. During the course of your participation in this study, if there is indication that you may pose a threat to yourself, you will be contacted by a licensed, clinical member of the research staff or be asked to visit the VA emergency department. This information will be handled under the same privacy and confidentiality standards as the rest of your research data, unless it is determined that immediate medical or mental health attention is required. Research staff is legally required to report known reasonable suspicion of abuse to a child, elder, or disabled adult. Staff are also legally required to report serious threats of physical violence against a reasonably identifiable victim or victims to law enforcement and the victim(s). Your information or biospecimens collected as part of the research, even if identifiers are removed, will not be used or distributed for future research studies.

A description of this clinical trial will be available on http://www.ClinicalTrials.gov as required by U.S. Law. This website will not include information that can identify you. At most, the website will include a summary of the results. You can search this website at any time. Any presentations or publications from this information will not identify you.

While this study is being conducted, you will not have access to your research related health records. This will not affect your VA healthcare including your doctor's ability to see your records as part of your normal care and will not affect your right to have access to the research records after the study is completed.

***AGREEMENT TO PARTICIPATE IN THE RESEARCH STUDY***

You have been informed that you do not have to take part in this study, and your refusal to participate will involve no penalty or loss of rights to which you are entitled. You may withdraw from this study at any time without penalty or loss of VA or other benefits to which you are entitled.

The study coordinator has explained the study to me. I have been told of the risks or discomforts and possible benefits of the study. I have been told of other choices of treatment available to me. I have been given the chance to ask questions and obtain answers.

By signing this document below, I voluntarily consent to participate in this study. I also confirm that I have read this consent, or it has been read to me. I will receive a copy of this consent after I sign it.

**I agree to participate in this research study as has been explained in this document.**

________________________________________ _________________________

Participant’s Signature Date

____________________________________ ______________________________ ______________

Signature of Researcher obtaining consent Name (print) Date

| **Health Information Portability and Accountability Act (HIPAA)** |
| --- |

There are rules to protect your private health information. Federal and state laws and the federal medical law, known as the HIPAA Privacy Rule, also protect your privacy. By signing this document, you provide your permission called your ‘authorization,’ for the access, use, and disclosure of information protected by the HIPAA Privacy Rule.

The research team working on the study will collect and use information learned from the procedures described in this consent form. They may also collect other information including your name, address, date of birth, and information from your medical records such as medical history, history of alcohol abuse, and mental health treatment.

The research team may also need to share your health information and the information it collects to other entities as part of the study progress. Other VA entities may include the VA Office of Research Oversight (ORO). You also give your permission for the research team to disclose your information to the Institutional Review Board, Food and Drug Administration (FDA), Office of Human Research Protections (OHRP), and the Government Accountability Office (GAO).

Your health information disclosed outside the VA as described in this authorization may no longer be protected by Federal laws or regulations and may be subject to re-disclosure by the recipient.

You can revoke this authorization, in writing, at any time. To revoke your authorization, you may (a) write to the Release of Information Office at this facility; (b) ask a member of the research team to give you a form to revoke the authorization; or (c) send your written request to the Principal Investigator for this study at the following address:

Jessica Bomyea and Andrea Spadoni Townsend

3350 La Jolla Village Dr, San Diego, CA 92161

MC 151b

If you revoke this authorization, Jessica Bomyea and Andrea Spadoni Townsend and their research team can continue to use information about you that was collected before receipt of the revocation. The research team will not collect information about you after you revoke the authorization.

While this study is being conducted you will not have access to your research-related health records.

Treatment, payment or enrollment/eligibility for benefits cannot be conditioned on you signing this authorization.

Unless you revoke (take back) your permission, your authorization to allow us to use and/or disclose

your information will expire at the end of this research study.

***AGREEMENT TO AUTHORIZE USE AND RELEASE OF INDIVIDUALLY IDENTIFIABLE HEALTH INFORMATION***

By signing this document below, I give my authorization (permission) for the use and disclosure of my individually identifiable health information as described in this document. This authorization has been explained to me and I have been given the opportunity to ask questions. If I believe that my privacy rights have been compromised, I may contact the VHA facility Privacy Officer to file a verbal or written complaint. I will be given a signed copy of this document for my records.

___________________________________ _____________ ___________________

Participant’s Signature Last 4 of SSN Date

| **EXPERIMENTAL SUBJECT’S BILL OF RIGHTS** |
| --- |

You have been asked to participate as a subject in medical research.

You have the right to know:

1. The nature and purpose of the study.
2. The procedures in the study and any drug or device to be used.
3. Discomforts and risks reasonably to be expected from the study.
4. Benefits reasonably to be expected from the study.
5. Alternative procedures, drugs, or devices that might be helpful to you and their risks and benefits.
6. Availability of medical treatment should complications occur.
7. You may ask questions about the study or the procedure.
8. You may quit the study at any time without affecting your future care at the VA.
9. You should be given a copy of the signed and dated written consent form for the study.
10. Your consent to participate must be given freely, without being obtained through deceit, force, or coercion.

If you have any questions or concerns about your rights as a research subject please contact the VASDHS Research Compliance Officer at (858) 642-3817 or RCO@vapop.ucsd.edu. You may leave an anonymous comment at the VASDHS research compliance hotline at 858-642-6311.

REF: California HSC 24170-24179.5
